# Post-MI: unsupervised brain tissue segmentation via post-maximized mutual information

**DOI:** 10.3389/fmed.2026.1852159

**Published:** 2026-08-04

**Authors:** Ke Niu, Jiadong Guo, Ming Zhang, Zhongmin Guo, Jiuyun Cai, Naian Xiao, Bin Jiang, Heng Miao

**Affiliations:** 1 Computer School, Beijing Information Science and Technology University, Beijing, China; 2 Department of Pediatrics, Zhongshan Hospital Xiamen University, Xiamen, Fujian, China; 3 Department of Neurology, The Third Hospital of Xiamen, Xiamen, Fujian, China; 4 Department of Neurology and Geriatrics, Fujian Institute of Geriatrics, Fujian Medical University Union Hospital, Fuzhou, Fujian, China; 5 Department of Neurology and Department of Neuroscience, The First Affiliated Hospital of Xiamen University, School of Medicine, Xiamen University, Xiamen, Fujian, China; 6 Xiamen Medical Quality Control Center for Neurology, Xiamen, Fujian, China; 7 Department of Ophthalmology, Peking University People's Hospital, Beijing, China; 8 Beijing Key Laboratory of Ocular Disease and Optometry Science, Peking University People's Hospital, Beijing, China

**Keywords:** age-related neurological conditions, aging-related neuroimaging, annotation-scarce learning, brain MRI, brain tissue segmentation, mutual information, unsupervised segmentation

## Abstract

**Introduction:**

Brain tissue segmentation in magnetic resonance imaging (MRI) is a fundamental step in quantitative neuroimaging and supports the analysis of structural brain alterations associated with aging. However, voxel-level annotation is expensive and labor-intensive, which limits the development of artificial intelligence methods in annotation-scarce neuroimaging settings. Existing mutual-information-based unsupervised segmentation methods mainly model consistency across different views of the same sample, while the representation derived from draft segmentation remains underexplored for mutual information modeling.

**Methods:**

In this paper, we propose Post-MI, an unsupervised brain tissue segmentation method that estimates mutual information between deep features extracted from the raw image and deep features derived from the probability map of the draft segmentation. A post-training strategy is introduced to enable this objective to refine the segmentation network after draft segmentation generation. In addition, edge information and a discriminative loss are incorporated to enhance boundary preservation and inter-class separability. We evaluate the proposed method primarily on IBSR-18 and further conduct exploratory experiments on BraTS2018.

**Results:**

On IBSR-18, Post-MI achieves a mean intersection over union of 0.3635 and a mean Dice score of 0.4661, outperforming the compared unsupervised baselines. Ablation experiments further verify the positive contributions of edge information and discriminative loss. On BraTS2018, exploratory results indicate that more complex tumor structures and weaker boundaries remain challenging for the current method.

**Discussion:**

Post-MI provides a feasible solution for brain MRI segmentation under annotation-scarce conditions and may facilitate quantitative structural brain analysis in aging-related neuroimaging research and other studies of age-related neurological conditions where large-scale manual delineation is difficult to obtain.

## 1 Introduction

Brain magnetic resonance imaging (MRI) is widely used to characterize structural brain changes associated with aging and age-related neurological disorders. Accurate segmentation of brain tissues, including gray matter, white matter, and cerebrospinal fluid, is a fundamental step in quantitative neuroimaging analysis and supports the assessment of brain atrophy, tissue composition, and other structural alterations that are relevant to studies of older adults. In recent years, supervised deep learning (1–4) has achieved state-of-the-art performance in medical image segmentation when large labeled datasets are available. Recent reviews, benchmark studies, and foundation-model advances have further accelerated progress in medical image segmentation (5–7). However, obtaining voxel-level annotations for brain MRI remains labor-intensive and expensive, because it requires experts with domain knowledge to provide time-consuming manual delineations. Therefore, developing segmentation methods that reduce reliance on labeled training data remains important for brain MRI analysis in aging-related neuroimaging research and age-related neurological conditions. Recent reviews of label-efficient segmentation and ultra-low-data learning have further underscored the practical importance of reducing annotation dependence in medical imaging (8–10). At the same time, universal segmentation models have begun to extend transferable priors across diverse medical imaging tasks (11, 12). This issue is particularly relevant in brain MRI, where recent studies and reviews continue to emphasize robust tissue and tumor segmentation across ages and disease settings (13, 14).

To reduce the reliance on manual annotation, a variety of unsupervised image segmentation methods have been investigated in recent years, among which mutual-information-based approaches constitute an important research direction (15–18). Related recent annotation-efficient medical image segmentation studies have further enriched this landscape through contrastive learning, pseudo-label refinement, and diffusion-based regularization (19–21). Ji et al. (15) estimated mutual information between predictions from different views of the same sample. Ouali et al. (16) modeled mutual information between predictions generated from two valid autoregressive orderings by incorporating masked convolutions into the network. Mirsadeghi et al. (17) further introduced adversarial regularization to stabilize representation learning in addition to mutual information maximization. Harb and Knöbelreiter (18) maximized mutual information between local features and class-level high-level features. Despite their effectiveness, these methods mainly improve feature learning before the generation of the draft segmentation probability map, while the representation derived from the draft segmentation itself remains insufficiently explored for mutual information modeling. Beyond MI-based methods, recent work has also emphasized robust representation learning through hard-positive contrastive learning, multi-modal translation, and prototype-based teacher-student training (22–24).

For further clarification, we transform the feature maps into logarithmic distributions and estimate the corresponding MI values for several open-source unsupervised MI-based approaches. Our empirical analysis shows that the proposed Post-MI strategy achieves a higher MI value than the compared methods. This finding provides strong empirical motivation for estimating mutual information between deep features extracted from the raw image and those derived from the probability map of the draft segmentation, which may capture a more informative feature relationship for unsupervised segmentation. Based on this motivation, we propose Post-MI, an unsupervised brain tissue segmentation method that estimates mutual information between these deep features.

In Post-MI, mutual information is estimated between two deep features, with one extracted from the raw image and the other derived from the probability map of the draft segmentation, both corresponding to the same input sample. In addition, we introduce a post-training strategy to optimize the segmentation process after draft segmentation generation. To alleviate the loss of boundary-related details in MI-based optimization, we further incorporate edge information extracted from the raw image (25). Moreover, to improve inter-class separability and intra-class compactness, we adapt a discriminative loss (26) to the unsupervised segmentation setting. Experimental observations show that the combination of edge information and discriminative loss leads to better performance.

The contributions of this paper are summarized as follows:

- We propose Post-MI, an unsupervised brain tissue segmentation method that estimates mutual information between deep features extracted from the raw image and those derived from the draft segmentation probability map.
- We introduce a post-training strategy that allows the mutual information objective to optimize the segmentation process after draft segmentation generation.
- We incorporate edge information and discriminative loss to enhance boundary preservation and improve inter-class separability in unsupervised brain tissue segmentation.
- We validate the proposed method primarily on IBSR-18 and further conduct exploratory experiments on BraTS2018 to analyze its performance in a more challenging tumor segmentation setting.

## 2 Related work

### 2.1 Unsupervised semantic segmentation

In supervised medical image segmentation, representative architectures such as V-Net have demonstrated the effectiveness of end-to-end volumetric learning, while DeepMedic and subsequent multi-scale 3D CNN models have shown strong performance in brain lesion and tumor segmentation tasks (27–29). In contrast, unsupervised semantic segmentation aims to perform segmentation without manual annotations and remains a highly challenging task. Existing methods can be broadly categorized into several groups. Other recent methods have explored consistency-driven optimization to stabilize training under limited annotations (30). Van Gansbeke et al. (31) proposed a two-step framework based on object mask proposals, but their method relies on pretraining on large-scale datasets and is therefore not fully unsupervised. W-Net (32) estimates segmentation maps from input images and reconstructs the original images from the estimated segmentations, although the reconstructed branch may introduce irrelevant information. Kim et al. (33) reformulated conventional clustering as a differentiable objective for segmentation, and Yin et al. (34) adopted a related idea by introducing a differentiable level-set loss.

Another line of research combines traditional algorithms with deep learning models (35–39). For example, Zhang et al. (36) first generated pseudo masks and then optimized the segmentation model in an iterative learning manner. Mutual-information-based approaches (15, 16) provide another important direction for unsupervised segmentation by maximizing MI between different views of the same instance at corresponding spatial locations. In contrast to these methods, our work focuses on modeling a more informative feature relationship between the raw image and the draft segmentation. Related studies in visually ambiguous scene understanding have also explored dynamic pseudo-label learning and edge-semantic collaboration to improve supervision efficiency and boundary-aware representation learning (40, 41).

### 2.2 Mutual information maximization

Mutual information (MI) is a fundamental measure of statistical dependence and has been widely applied in information theory, statistics, and machine learning. In unsupervised segmentation, IIC (15) maximizes MI to enforce consistent predictions for differently augmented image patches. Ouali et al. (16) extended this idea by introducing masked convolutions in addition to geometric transformations. InMARS (17) incorporated adversarial regularization into MI-based segmentation, while InfoSeg (18) maximized MI between local features and high-level semantic features from a different perspective.

To address the difficulty of estimating MI for high-dimensional continuous random variables, several studies have proposed neural lower-bound estimators (42, 43). In addition, MI has been widely explored in unsupervised representation learning (42, 44, 45). Inspired by these studies, our method estimates MI between deep features extracted from the raw image and those derived from the draft segmentation, which is more directly aligned with the segmentation process in our setting.

## 3 Motivation

**Figure 1:**
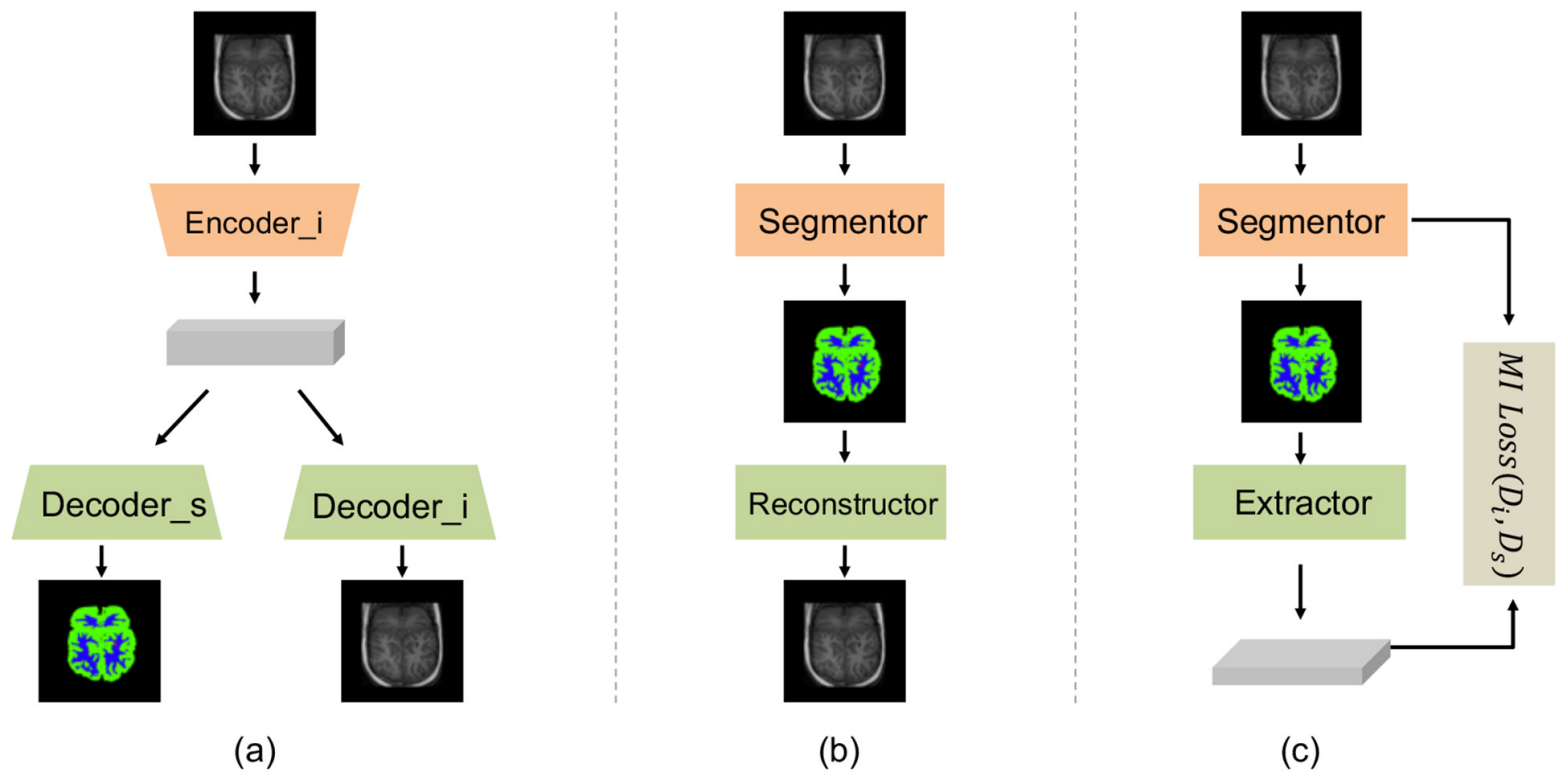
**(a)** An intermediate latent representation can support both segmentation and image reconstruction, suggesting that it preserves information relevant to both tasks. **(b)** The raw image is first transformed into a segmentation map and then reconstructed back to the image domain, indicating a close relationship between image representations and segmentation representations. **(c)** *D*_*i*_ denotes the deep features extracted from the raw image in the Segmentor, and *D*_*s*_ denotes the deep features extracted from the draft segmentation probability map in the Extractor.

Before presenting the proposed method, we first describe its motivation. As illustrated in Figure 1, prior studies suggest that intermediate representations between the input image and the segmentation output may retain information from both domains. In Figure 1a, the latent representation produced by Encoder_i is used for both segmentation and image reconstruction, indicating that this intermediate representation contains information relevant to both tasks. For example, Myronenko (46) introduced a VAE branch to reconstruct the input image in a brain tumor segmentation architecture. In Figure 1b, the raw image is first transformed into a segmentation map and then reconstructed back to the original image, suggesting a strong relationship between image representations and segmentation representations. W-Net (32) is a representative example of this idea.

These observations imply that the features derived from the raw image and those derived from the draft segmentation should share informative structure. At the same time, excessively deep latent representations may suffer from information loss. Therefore, instead of directly operating on the final segmentation output, we choose to estimate mutual information between deep features extracted from the raw image and those derived from the draft segmentation. The resulting architecture is illustrated in Figure 1c.

## 4 Method

### 4.1 Analysis of deep features

To analyze the role of deep features, we take U-Net (47) as an illustrative example. U-Net consists of three main parts, namely the encoder, the decoder, and the latent representation. By visualizing the features from different layers in a segmentation setting, we observe that shallow features are more similar to the input image, whereas deeper decoder features are closer to the segmentation output. In contrast, the latent representation located between the encoder and decoder serves as an intermediate feature space, as discussed in Section 3.

In our model, the architecture is simplified to include only one downsampling stage and one upsampling stage. Under this design, the extracted deep feature is conceptually closer to the latent representation. Therefore, we estimate mutual information between the deep features of the input image and those derived from the draft segmentation, rather than between the input image and the final segmentation output, to guide model optimization.

### 4.2 Overview

**Figure 2:**
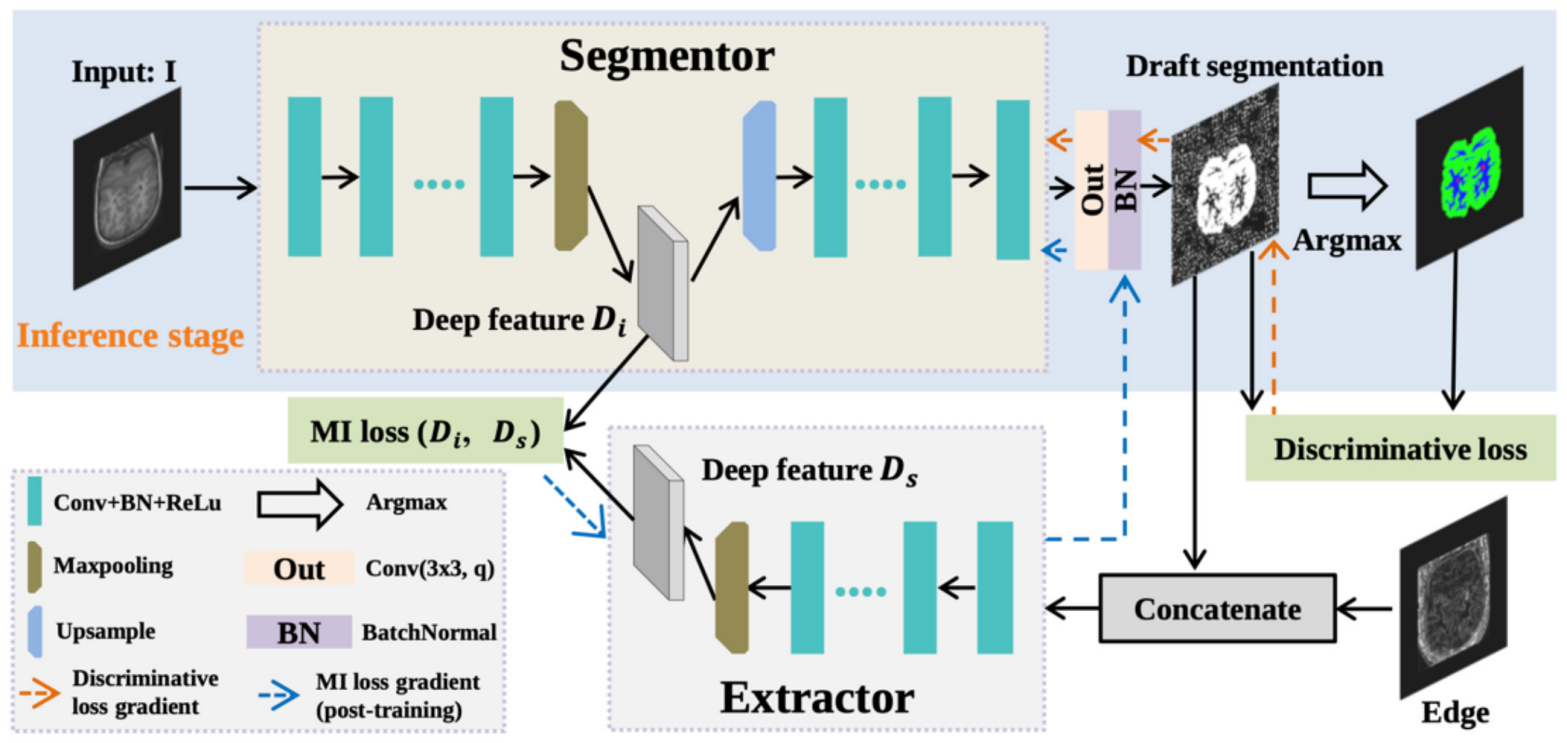
The training stage of the Post-MI method. The dashed lines indicate the directions of gradient backpropagation. During training, *D*_*i*_ denotes the deep features extracted from the raw image, and *D*_*s*_ denotes the deep features derived from the draft segmentation. The *argmax* operation is used only to generate pseudo-label assignments for the discriminative loss and is not involved in gradient propagation. During inference, the Extractor is discarded and the Segmentor produces the final segmentation. Here, *q* denotes the number of classes, and each unique cluster is assigned a label between 1 and *q*.

Figure 2 illustrates the overall architecture of the proposed Post-MI network. The model is trained by estimating mutual information between two deep features, one extracted from the raw image and the other derived from the draft segmentation. The dashed lines indicate the directions of gradient backpropagation during training. The proposed network consists of two components, namely the Segmentor and the Extractor, both of which are composed of convolution, normalization, and activation layers.

During training, the Segmentor generates a draft segmentation and simultaneously produces the deep feature *D*_*i*_ from the raw image for mutual information estimation. The draft segmentation is represented in a *q*-dimensional space, where *q* denotes the number of cluster labels. The Extractor takes the concatenation of the draft segmentation and the edge information as input and produces another deep feature *D*_*s*_ for mutual information estimation. Since *D*_*s*_ is derived from a representation located after the draft segmentation, we introduce a post-training strategy to compute the mutual information loss and backpropagate the corresponding gradients through both the Extractor and the Segmentor, as indicated by the blue dashed line.

In parallel, after the draft segmentation is generated, pseudo-label assignments are obtained by applying the *argmax* operation to the draft segmentation output at each spatial location. These pseudo-label assignments are used only to define cluster memberships for the discriminative loss. Since the *argmax* operation is non-differentiable, it is treated as a fixed assignment step and does not receive gradients. The discriminative loss is computed with respect to the continuous segmentation representation and its gradients are backpropagated only through the Segmentor, as indicated by the orange dashed line. During inference, the Extractor is discarded, and the final segmentation result is produced solely by the Segmentor.

### 4.3 Post-maximizing mutual information

As discussed in Sections 3, 4.1, we adopt a post-training strategy to estimate mutual information (MI) between two deep features. We first recall the definition of MI between two random variables *X* and *Y* as follows:


$$\begin{array}{l} I\left(X;Y\right)=H\left(X\right)-H\left(X|Y\right) \end{array}$$ (1)

where *H*(·) denotes entropy. This definition characterizes the amount of shared information between two random variables. In our method, *X* and *Y* correspond to *D*_*i*_ and *D*_*s*_, respectively, where *D*_*i*_ denotes the deep features extracted from the raw image and *D*_*s*_ denotes the deep features derived from the draft segmentation. Accordingly, Equation 1 can be rewritten for our setting as:


$$\begin{array}{l} I\left(D_{i};D_{s}\right)=H\left(D_{i}\right)-H\left(D_{i}|D_{s}\right) \end{array}$$ (2)

The objective is therefore to maximize *I*(*D*_*i*_; *D*_*s*_). Here, *D*_*i*_ is obtained from *f*(*x*) and *D*_*s*_ is obtained from *E*(*S*(*x*)), where *x* is the raw image, *f*(·) denotes the deep-feature generator for the raw image, *E*(·) denotes the Extractor, and *S*(·) denotes the Segmentor.

Since the deep features from the raw image and the draft segmentation are high-dimensional continuous random variables, direct MI computation is challenging. To address this issue, we adopt MINE (43), which estimates MI by maximizing a neural lower bound. Specifically, the JSD-based MI estimator for two random variables *X* and *Y* (48) is defined in Equation 3 as follows:


$$\begin{array}{r} I\left(X;Y\right)\geq {Î}_{JSD}\left(X;Y\right)=E_{p\left(x,y\right)}\left[-sp\left(-T\left(x,y\right)\right)\right]\\ -E_{p\left(x\right)p\left(y\right)}\left[sp\left(T\left(x,y\right)\right)\right] \end{array}$$ (3)

where *sp*(*x*) = log(1+*e*^*x*^), and *T* is a discriminator implemented as a fully connected layer that maps sample pairs from *X* and *Y* to a real-valued score. Here, *p*(*x, y*) denotes the joint distribution, while *p*(*x*) and *p*(*y*) denote the corresponding marginal distributions. Applied to our setting, the estimator becomes:


$$\begin{array}{r} {Î}_{JSD}\left(D_{i};D_{s}\right)=E_{p}\left[-sp\left(-T\left(\left[D_{i};D_{s}\right]\right)\right)\right]\\ -E_{p\left(D_{i}\right)p\left(D_{s}\right)}\left[sp\left(T\left(\left[D_{i};D_{s}\right]\right)\right)\right] \end{array}$$ (4)

where *p* denotes the empirical data distribution. The estimator in Equation 4 is used to approximate the mutual information objective in Equation 2. In implementation, we maximize this estimator by minimizing its negative form, as defined in Section 4.5.

### 4.4 Edge supplemented and discriminative loss

#### 4.4.1 Edge supplement

**Figure 3:**
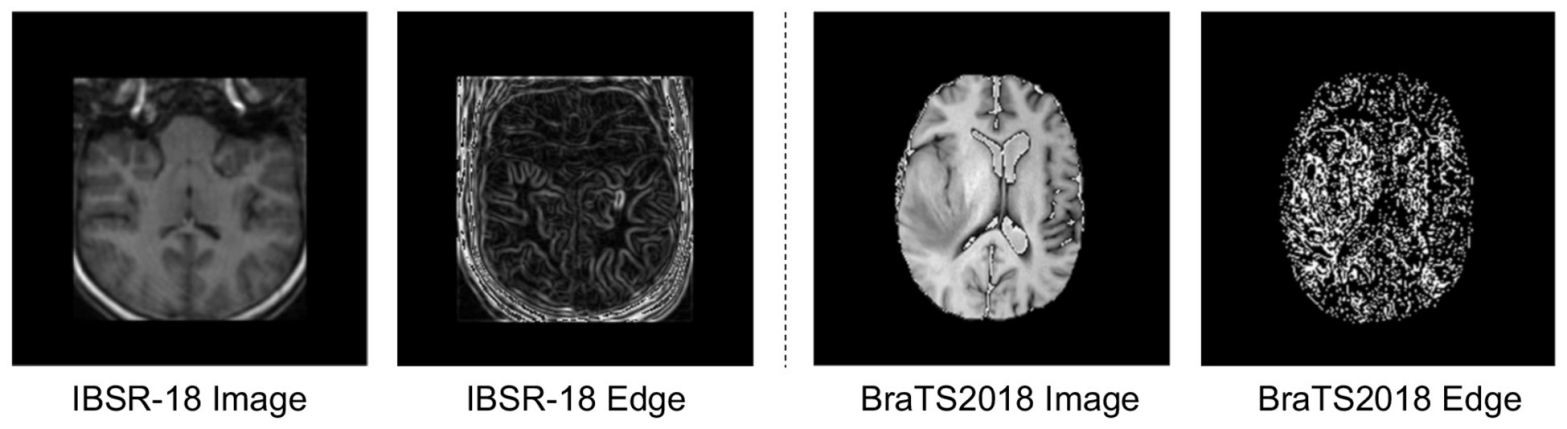
Representative MRI images and corresponding edge maps from IBSR-18 and BraTS2018. Compared with BraTS2018, IBSR-18 exhibits relatively clearer structural boundaries.

To alleviate the loss of low-level boundary information in mutual-information-based optimization, we extract edge information from the raw image to provide additional boundary cues. Figure 3 shows representative MRI images and corresponding edge maps. The IBSR-18 example is used to illustrate the edge information used in our method, while the BraTS2018 example is included for comparison. During training, the edge map is concatenated with the draft segmentation and fed into the Extractor, so that the resulting mutual information loss can guide the Segmentor while reducing the influence of irrelevant information. The Sobel filters used to generate the edge map are defined in Equation 5 as follows:


$$\begin{array}{l} G_{x}=\left[\begin{array}{ccc} -1 & -2 & -1\\ 0 & 0 & 0\\ 1 & 2 & 1 \end{array}\right]*A\;\text{and }G_{y}=\left[\begin{array}{ccc} -1 & 0 & 1\\ -2 & 0 & 2\\ -1 & 0 & 1 \end{array}\right]*A \end{array}$$ (5)

Here, *G*_*x*_ and *G*_*y*_ denote the horizontal and vertical gradient responses, respectively, and *A* denotes the raw image. The final edge map is computed according to Equation 6 as follows:


$$\begin{array}{l} Edge=\sqrt{{\left(G_{x}\right)}^{2}+{\left(G_{y}\right)}^{2}} \end{array}$$ (6)

As shown in Figure 3, the IBSR-18 edge map provides clearer boundary information for the subsequent mutual information estimation.

#### 4.4.2 Discriminative loss

In addition to edge information, we introduce an auxiliary discriminative loss (26) to improve inter-class separability and intra-class compactness in our approach. For brevity, we denote the discriminative loss by *L*_*disc*_ hereafter. It consists of three terms: variance, distance, and regularization. We adapt the discriminative loss to the unsupervised setting. The loss function is as follows:


$$\begin{array}{l} L_{var}=\frac{1}{C}\overset{C}{\underset{c=1}{\sum }}\frac{1}{N_{c}}\overset{N_{c}}{\underset{i=1}{\sum }}{\left[||u_{c}-x_{i}||-\delta_{v}\right]}_{+}^{2} \end{array}$$ (7)

$$\begin{array}{l} L_{dist}=\frac{1}{C\left(C-1\right)}\overset{C}{\underset{c_{A}=1}{\sum }}\overset{C}{\underset{c_{B}=1}{\sum }}{\left[2\delta_{d}-||u_{c_{A}}-u_{c_{B}}||\right]}_{+}^{2}\;\left(c_{A}\neq c_{B}\right) \end{array}$$ (8)

$$\begin{array}{l} L_{reg}=\frac{1}{C}\overset{C}{\underset{c=1}{\sum }}||u_{c}|| \end{array}$$ (9)

$$\begin{array}{l} y=argmax\left(x\right) \end{array}$$ (10)

$$\begin{array}{l} L_{disc}=\alpha \cdot L_{var}+\beta \cdot L_{dist}+\gamma \cdot L_{reg} \end{array}$$ (11)

In Equations 7–11, *C* denotes the number of clusters, *x* denotes the embedding of the segmentation output, *N*_*c*_ is the number of elements in cluster *c*, and *u*_*c*_ is the mean embedding of cluster *c* (i.e., the cluster center). The operator *argmax*(*x*) denotes the pseudo-label assignment derived from the segmentation output and is used only to group pixels into clusters when computing the discriminative loss. The *argmax* operation is not involved in gradient computation. During optimization, the discriminative loss is differentiated with respect to the continuous segmentation representation *x*, and the resulting gradients are backpropagated to the Segmentor through *x*. ||·|| denotes the *L*_1_ or *L*_2_ distance, and [·]_+_ = max(0, ·) denotes the hinge function. δ_*v*_ and δ_*d*_ are the margins for the variance and distance terms, respectively. In our experiments, we set δ_*v*_ = 0, δ_*d*_ = 1.5, α = 1, β = 1, and γ = 0.001. These hyperparameters were chosen empirically with reference to prior discriminative-loss studies (26, 49) and were fixed across all experiments.

### 4.5 Loss function

We optimize the proposed model using two loss terms. The first is the mutual information loss, which encourages consistency between the deep features extracted from the raw image and those derived from the draft segmentation. The second is the discriminative loss, which improves inter-class separability and intra-class compactness. These two loss terms are complementary in the training process. Since the objective in Section 4.3 is to maximize the mutual information between *D*_*i*_ and *D*_*s*_, in practice we minimize the negative JSD-based estimator. The mutual information loss is defined in Equation 12, and the overall loss function is defined in Equation 13, as follows:


$$\begin{array}{l} L_{MI}=-{Î}_{JSD}\left(D_{i};D_{s}\right) \end{array}$$ (12)

$$\begin{array}{l} L_{total}=a\cdot L_{MI}+b\cdot L_{disc} \end{array}$$ (13)

Here, *L*_*MI*_ denotes the negative form of the JSD-based mutual information estimator in Equation 4. Minimizing *L*_*MI*_ is therefore equivalent to maximizing the estimated mutual information between *D*_*i*_ and *D*_*s*_. In our experiments, we set *a* = 1 and *b* = 1.

## 5 Experiments and results

To assess the performance of the proposed Post-MI method, we carry out experiments on the IBSR-18 (50) and BraTS2018 (51) datasets. In this section, we briefly describe datasets and evaluation metrics as well as give implementation details for our method.

### 5.1 Datasets

The IBSR-18 dataset (50) is a brain tissue segmentation dataset consisting of 18 T1-weighted MRI brain volumes of size 256 × 128 × 256. The manual annotations provide four labels, corresponding to background, cerebrospinal fluid (CSF), gray matter (GM), and white matter (WM), respectively. In our experiments, each MRI volume was processed as 128 axial slices of size 256 × 256. Following axial slicing, we adopted a fixed slice-level split for all experiments. Specifically, 270 slice–mask pairs were reserved as the test set, and the remaining slices were divided into the training and validation sets at a ratio of 8:2. The same fixed split was used for all compared methods throughout the experiments.

The BraTS2018 dataset (51) provides multimodal brain MRI scans, including T1-weighted, post-contrast T1-weighted, T2-weighted, and FLAIR images. The tumor annotations include edema, necrotic and non-enhancing tumor, and enhancing tumor regions. In this study, we conduct two exploratory experiments on BraTS2018 using T1-weighted images: (1) binary segmentation for whole tumor segmentation, and (2) multi-class segmentation for whole tumor (WT), enhanced tumor (ET), and tumor core (TC).

### 5.2 Evaluation metric

We evaluated the proposed method on 270 test slices from IBSR-18 and 270 test slices from BraTS2018. In this study, Intersection over Union (IoU) and the Dice coefficient were adopted as the evaluation metrics.

### 5.3 The details of computation for metrics

The model produces *q* clusters, and each cluster is assigned to a target class through an index-matching process. Specifically, for each target class, we first identify the cluster index with the highest score and then remove this index from the candidate set to avoid duplicate assignment. This procedure is repeated until all target classes have been assigned unique cluster indices. Let *l*_*index*_ denote the list of candidate cluster indices. The matching procedure is summarized as follows:

(a) Find the index *i* with the highest score for the current target class in *l*_*index*_.

(b) Save *i* and remove it from *l*_*index*_.

(c) For the next target class, search for the index with the highest score in the remaining *l*_*index*_.

(d) Repeat steps (a)–(c) until all target classes have been assigned unique indices.

This matching procedure is performed independently for each test slice rather than per subject or over the whole test set. After the cluster-to-class assignment is determined for a slice, IoU and Dice are computed on that slice, and the final reported values are obtained by averaging the per-slice scores over all test slices.

### 5.4 Experimental details

In our experiments, the model was trained using the Adam optimizer with a learning rate of 0.0003. Each input was an axial MRI slice of size 256 × 256. Before being fed into the network, the slice intensities were normalized. No additional preprocessing, such as bias field correction, was applied. The maximum number of training epochs was set to 10,000, and an early stopping strategy with a patience of 100 epochs was adopted to terminate training. The overall loss function is defined in Equation 13. We set *a* = 1 and *b* = 1 in Equation 13, and set δ_*v*_ = 0, δ_*d*_ = 1.5, α = 1, β = 1, and γ = 0.001 in Equation 11.

### 5.5 Baseline

To evaluate the proposed method, we compare Post-MI with several representative unsupervised segmentation approaches, including K-means clustering, IIC (15), and the autoregressive unsupervised image segmentation method of Ouali et al. (16), denoted as AC in the following text and tables. We also include two related unsupervised segmentation methods, namely Kim et al. (33) and Kanezaki (35), for further comparison. For the learning-based baselines, we re-implemented the compared methods under the same backbone architecture as the Segmentor shown in Figure 2 to reduce architectural bias in the comparison.

### 5.6 Results

In this section, we present the results of our method on brain tissue segmentation (IBSR-18) and brain tumor segmentation (BraTS2018).

#### 5.6.1 Brain tissue segmentation (IBSR-18)

Table 1 presents the results of brain tissue segmentation on the IBSR-18 dataset for the GM, WM, and CSF categories. Here, Mean IoU and Mean Dice are computed as the averages over the three tissue classes, i.e., Mean IoU = (GM IoU + WM IoU + CSF IoU)/3 and Mean Dice = (GM Dice + WM Dice + CSF Dice)/3, and are therefore regarded as the primary evaluation metrics. IIC(Mine) + MI denotes the IIC variant that computes mutual information between the image and the segmentation output. As shown in Table 1, Post-MI achieves a Mean IoU of 0.3635 and a Mean Dice score of 0.4661, outperforming the compared baseline methods. These results suggest that estimating mutual information between two deep features is more effective than directly computing it between the image and the segmentation output. In addition, the comparison between Post-MI and Post-MI(w/skip) indicates that incorporating edge information is more beneficial than introducing skip connections for supplementing low-level information in this setting.

**Table 1:** Comparisons of IoU and Dice coefficients for unsupervised brain tissue segmentation on IBSR-18.

Method	GM IoU	WM IoU	CSF IoU	Mean IoU	GM Dice	WM Dice	CSF Dice	Mean Dice
K-means	0.3225	0.1823	0.0061	0.1703	0.4603	0.2689	0.0145	0.2479
AC	0.4162	0.0392	0.0010	0.1522	0.5877	0.0754	0.0019	0.2641
IIC (Mine)	0.3817	0.3317	0.0289	0.2474	0.5425	0.4884	0.0552	0.3620
Kim et al.	0.2686	0.2399	0.0215	0.1766	0.4164	0.3717	0.0408	0.2763
IIC (Mine) + MI	0.2547	0.1686	0.0181	0.1471	0.4011	0.2780	0.0351	0.2380
Post-MI(w/skip)	0.4042	0.5129	0.0108	0.3093	0.5643	0.6667	0.0211	0.4174
Post-MI	0.5264	0.5449	0.0193	0.3635	0.6836	0.6773	0.0373	0.4661

Higher values indicate better performance. Mean IoU = (GM IoU + WM IoU + CSF IoU)/3 and mean Dice = (GM Dice + WM Dice + CSF Dice)/3, and these two metrics are regarded as the primary evaluation metrics. Post-MI(w/skip) denotes the variant with a skip connection between the max-pooling and upsampling layers in the Segmentor shown in Figure 2. IIC (Mine) + MI denotes the IIC variant that computes mutual information between the image and the segmentation output.

**Figure 4:**
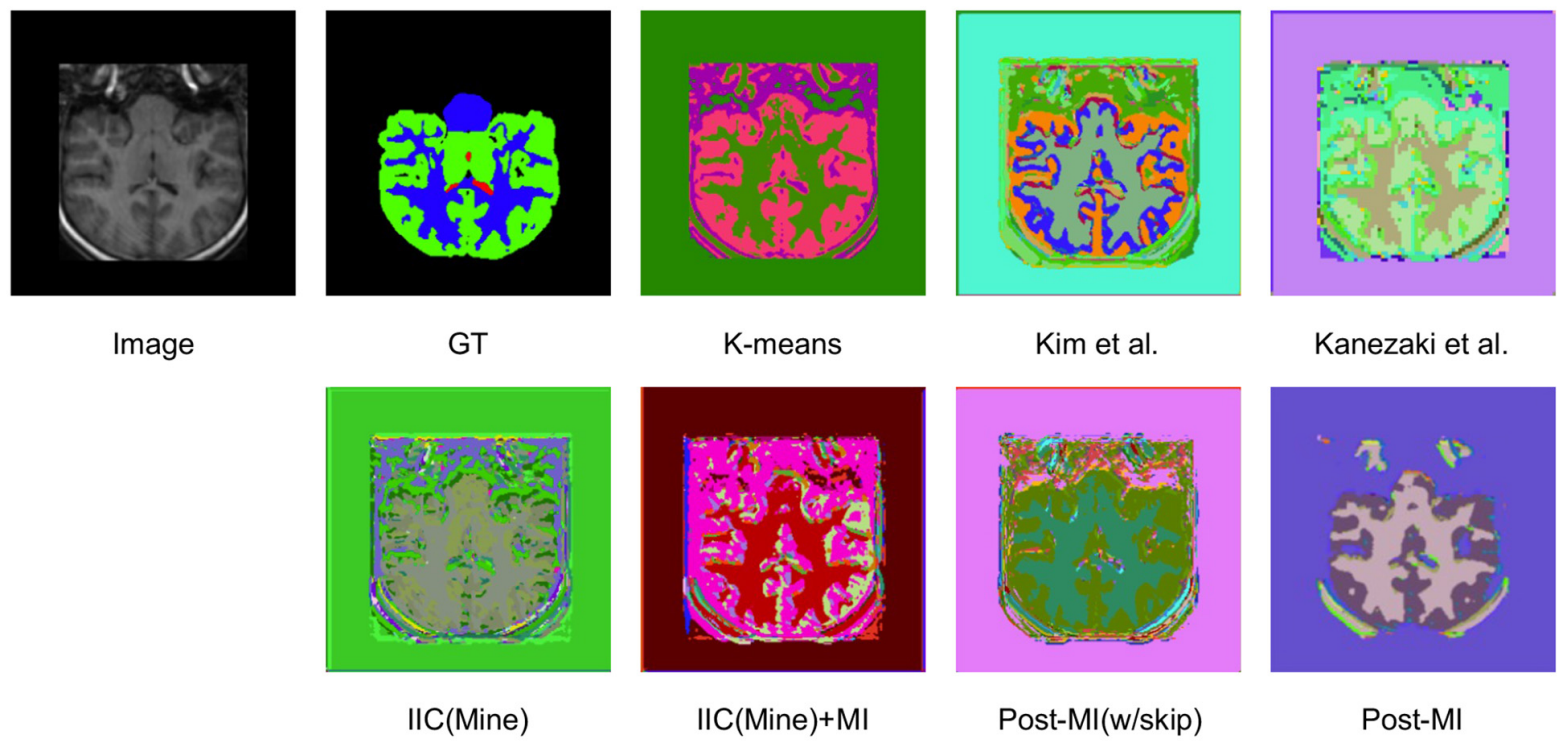
Qualitative visualization of brain tissue segmentation results on the IBSR-18 dataset. Different colors across the images represent different categories because the output clusters are randomly generated and then matched to the target classes. In the ground truth, black denotes background, green denotes GM, blue denotes WM, and red denotes CSF.

As shown in Figure 4, we further compare the qualitative segmentation results of different approaches on IBSR-18. Different colors in different images represent different categories because the output clusters are randomly generated and then matched to the target classes. Figure 4 shows that the K-means method tends to misclassify white matter as background in some cases, which degrades the segmentation quality. In contrast, Post-MI yields comparatively less scattered noise and clearer tissue boundaries than the other methods, which is consistent with its superior quantitative performance reported in Table 1.

#### 5.6.2 Ablation study on IBSR-18

To further validate the proposed design, we conducted an ablation study under the same experimental setting as in Section 5.6.1. For clarity, we use a unified naming rule throughout the following ablation analysis: “-a” denotes the edge-only variant, “-b” denotes the discriminative-loss-only variant, “-pure” denotes the variant without edge information or discriminative loss, and “Post-MI(w/skip)” denotes the skip variant. As shown in Table 2, we investigated the effects of edge information, discriminative loss, and backbone design (with or without skip connections).

**Table 2:** Ablation study of edge information and discriminative loss on IBSR-18.

Method	Skip	*L*_*disc*_	Edge	GM IoU	WM IoU	CSF IoU	Mean IoU	GM Dice	WM Dice	CSF Dice	Mean Dice
Post-MI(w/skip)-pure	✓			0.4915	0.4610	0.0324	0.3283	0.6497	0.6132	0.0603	0.4411
Post-MI(w/skip)-b	✓	✓		0.4613	0.4931	0.0186	0.3243	0.6238	0.6429	0.0358	0.4342
Post-MI(w/skip)-a	✓		✓	0.3657	0.1758	0.0120	0.1845	0.5256	0.2850	0.0231	0.2779
Post-MI(w/skip)	✓	✓	✓	0.4042	0.5129	0.0108	0.3093	0.5643	0.6667	0.0211	0.4174
Post-MI-pure				0.3905	0.4006	0.0348	0.2753	0.5584	0.5604	0.0652	0.3946
Post-MI-b		✓		0.5085	0.5500	0.0173	0.3586	0.6645	0.6944	0.0330	0.4640
Post-MI-a			✓	0.3566	0.1930	0.0019	0.1838	0.5168	0.3060	0.0036	0.2755
Post-MI		✓	✓	0.5264	0.5449	0.0193	0.3635	0.6836	0.6773	0.0373	0.4661

The entries “skip” and “*L*_*disc*_” denote the use of skip connections and discriminative loss, respectively. Post-MI(w/skip) denotes the variant with a skip connection between the max-pooling and upsampling layers in the Segmentor shown in Figure 2.

**Figure 5:**
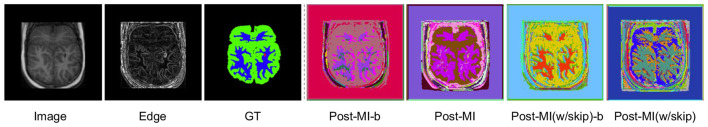
Qualitative comparison illustrating the effect of edge information. The variants with the suffix “-b” do not incorporate edge information to supplement low-level details. In the ground truth, black denotes background, green denotes GM, blue denotes WM, and red denotes CSF.

From the first four rows, the use of skip connections appears to reduce performance in this task, possibly because they introduce additional noise and do not cooperate well with edge information and discriminative loss. This observation is supported by the fact that Post-MI(w/skip) performs worse than Post-MI. In addition, considering the entire table, both Post-MI-a and Post-MI(w/skip)-a yield lower performance than the other variants, indicating that using edge information alone to supplement low-level details is insufficient and may introduce irrelevant information. In contrast, the combination of edge information and discriminative loss in Post-MI achieves the best overall performance, suggesting that these two components are more effective when used together. To provide a more intuitive comparison of the influence of edge information and skip connections, the corresponding qualitative results are further shown in Figure 5.

#### 5.6.3 Edge supplemented experiment on IBSR-18

As shown in Figure 5, we further examine the qualitative effect of edge information on IBSR-18 segmentation. Methods that incorporate edge information generally produce better segmentation results than their counterparts without edge supplementation, especially in the GM category. Figure 5 also shows that, compared with models without skip connections, the variants with skip connections exhibit more noise in the predicted regions. This observation is consistent with the ablation results in Table 2 and suggests that skip connections may interfere with the effective use of edge information in this model. Overall, Post-MI achieves the best qualitative performance in Figure 5.

#### 5.6.4 Exploratory analysis on BraTS2018

##### 5.6.4.1 Multi-class segmentation

Table 3 presents the exploratory results of unsupervised brain tumor segmentation on BraTS2018 for the WT, TC, and ET categories. Compared with the IBSR-18 brain tissue segmentation task, the BraTS2018 setting is substantially more challenging because of the higher structural complexity of tumors, the heterogeneous appearance across subregions, and the relatively low contrast of some boundaries in T1-weighted images. Under this challenging setting, the compared methods, including Post-MI, show less stable performance than on IBSR-18, indicating that unsupervised tumor segmentation on BraTS2018 remains difficult for the current method.

**Table 3:** Exploratory results of unsupervised multi-class segmentation on BraTS2018.

Method	WT IoU	TC IoU	ET IoU	Mean IoU	WT Dice	TC Dice	ET Dice	Mean Dice
K-means	0.0973	0.0133	0.0097	0.0401	0.2445	0.0731	0.0322	0.1166
AC	0.0309	0.0390	0.2878	0.1192	0.0590	0.0728	0.2878	0.1399
Kim et al.	0.1745	0.0998	0.0383	0.1042	0.2844	0.1698	0.0706	0.1749
Kanezaki et al.	0.1788	0.1061	0.0455	0.1101	0.2918	0.1800	0.0830	0.1849
IIC(Mine) + MI	0.0309	0.0367	0.2878	0.1185	0.0590	0.0686	0.2878	0.1385
Post-MI(w/skip)	0.0309	0.0448	0.2878	0.1212	0.0590	0.0827	0.2878	0.1431
Post-MI	0.1277	0.0639	0.0220	0.0712	0.2167	0.1140	0.0418	0.1241

Higher values indicate better performance for IoU and Dice. Mean IoU = (WT IoU + ET IoU + TC IoU)/3 and Mean Dice = (WT Dice + ET Dice + TC Dice)/3. Post-MI(w/skip) denotes the variant with a skip connection between the max-pooling and upsampling layers in the Segmentor shown in Figure 2. IIC(Mine) + MI indicates that the IIC(Mine) approach computes mutual information between the image and the segmentation output.

##### 5.6.4.2 Binary segmentation

We also conducted an exploratory binary segmentation experiment on BraTS2018, as summarized in Table 4. The overall performance remains limited compared with the IBSR-18 results. To further analyze this phenomenon, Figure 3 compares representative MRI images and edge maps from IBSR-18 and BraTS2018. Unlike the relatively clearer tissue boundaries in IBSR-18, tumor-related regions in BraTS2018 are often characterized by weak contrast, heterogeneous appearance, and gradual intensity transitions in T1-weighted images. Under such conditions, Sobel-based edge extraction tends to produce weak or fragmented boundary responses and may also capture irrelevant intensity variations from surrounding tissues. As a result, the extracted edge maps provide less reliable boundary cues for the proposed edge-supplemented design, which partly explains the limited performance of Post-MI on BraTS2018. These observations suggest that, although the proposed method is effective for unsupervised brain tissue segmentation, further methodological adaptation is needed for more complex tumor segmentation scenarios.

**Table 4:** Exploratory results of unsupervised binary segmentation on BraTS2018.

Method	IoU	Dice
K-means	0.0948	0.2366
AC	0.0924	0.1628
Kim et al.	0.1910	0.3064
Kanezaki et al.	0.1953	0.3162
IIC (Mine) + MI	0.1227	0.2097
Post-MI(w/skip)	0.1261	0.2136
Post-MI	0.1502	0.2425

Higher values indicate better performance for IoU and Dice.

## 6 Conclusion

In this paper, we present Post-MI, an unsupervised brain tissue segmentation method that maximizes mutual information between deep features derived from the input image and the draft segmentation. The proposed method introduces a post-training strategy to optimize the segmentation process and incorporates discriminative loss and edge information to alleviate the limitations of conventional MI-based optimization. Experimental results on the IBSR-18 benchmark demonstrate the effectiveness of the proposed method for unsupervised brain tissue segmentation. In addition, exploratory experiments on BraTS2018 indicate that the proposed method faces greater challenges in tumor segmentation scenarios with complex structures and low-contrast boundaries. These findings suggest that Post-MI is currently more suitable for relatively clearer brain tissue segmentation tasks, while future work should focus on improving its robustness in more complex pathological segmentation settings. Post-MI provides a feasible solution for unsupervised brain tissue segmentation and may support quantitative structural brain analysis in aging-related neuroimaging research and other studies of age-related neurological conditions. Overall, the present study demonstrates the promise of Post-MI for brain MRI analysis in aging-related and age-related neurological settings.

## Data Availability

The original contributions presented in the study are included in the article/supplementary material, further inquiries can be directed to the corresponding authors.
